# Transient Increase of Retinal Nerve Fiber Layer Thickness after Vitrectomy with ILM Peeling for Idiopathic Macular Hole

**DOI:** 10.1155/2016/5903452

**Published:** 2016-10-10

**Authors:** Kouichi Ohta, Atsuko Sato, Nami Senda, Emi Fukui

**Affiliations:** Department of Ophthalmology, Matsumoto Dental University, Nagano Prefecture, Japan

## Abstract

*Purpose*. The purpose of this study was to determine the long-term changes in the circumpapillary retinal nerve fiber layer (RNFL) thickness following macular hole surgery with internal limiting membrane (ILM) peeling combined with phacoemulsification.* Methods*. Thirty-eight eyes of 37 patients who had pars plana vitrectomy (*n* = 36) between 2010 and 2014 were studied. The average thicknesses of the global and the six sectors of the RNFL were determined before and at 1, 3, 6, 12, and 24 (*n* = 22) months (M) after the surgery by spectral-domain optical coherent tomography. The postoperative mean RNFL thickness at each time was compared to that before the surgery by paired* t*-tests.* Results*. The RNFL of the operated eyes was significantly thicker at 1 month (1 M) and 3 M in all but the inferior-nasal sectors. The significant increase remained until 12 M in the superior-temporal and superior-nasal sectors. In addition, the RNFL was also significantly thicker in the temporal-inferior sector at 12 M based on the findings in 38 eyes.* Conclusions*. The postoperative RNFL was thicker in all but the nasal-inferior sector for at least 12 M after surgery. This prolonged increase of the RNFL thickness may indicate damage and mild edema of the RNFL.

## 1. Introduction 

Kelly and Wendel reported that idiopathic macular holes (MHs) can be closed by pars plana vitrectomy (PPV) with fluid-gas exchange [1, 2]. Thereafter, the surgical techniques for closing a MH have been modified by removing the internal limiting membrane (ILM) which increased the anatomic success rates and improved the functional results [3–5]. Despite the increased success rates with ILM peeling, it is still debated whether ILM peeling is completely safe for the retina. It has been reported that there is an early transient swelling of the arcuate retinal nerve fiber layer (RNFL) and a later development of a dissociated optic nerve fiber layer (DONFL) appearance after ILM peeling [6, 7]. Although the DONFL appearance may not influence visual recovery or decrease the retinal sensitivity [6–9], the indocyanine green G (ICG) dye used to make the ILM more visible may do so [10–12].

The circumpapillary RNFL thickness can be measured by optical coherence tomography (OCT). A thinning of the RNFL and defects of the corresponding visual fields have been reported in MH patients who had undergone ICG-assisted ILM peeling [12]. Time-domain- (TD-) OCT was used to measure the thickness of the RNFL in these earlier studies, but the recently developed spectral-domain- (SD-) OCT processes data 50 times faster, is more sensitive, and can obtain higher resolution images. Quantification of the SD-OCT images showed a transient increase of the RNFL thickness 1 month after the ILM peeling [13–16]. However, the follow-up period was relatively short at 6 to 12 months.

At present, a prospective retinal and optic nerve vitrectomy evaluation (PROVE) study is on-going [17, 18]. However, the subjects being studied are not only those with a MH but also epiretinal membrane (ERM) and other retinal diseases. The published report was the results of only the 12-month findings [18].

The purpose of this study was to determine the long-term changes in the cpRNFL thickness in eyes with a MH that had undergone vitrectomy with ILM peeling. To accomplish this, we used SD-OCT with an eye tracking system to evaluate the changes in the cpRNFL thickness for 24 months in patients who underwent pars plana vitrectomy (PPV) with TA-assisted ILM peeling.

## 2. Methods

### 2.1. Patients

The medical charts of 38 eyes of 37 Japanese patients (17 men and 20 women) with a full-thickness MH who had undergone vitrectomy with triamcinolone acetonide- (TA-) assisted ILM peeling at the Matsumoto Dental University Hospital between February 2010 and July 2014 were reviewed (Table 1). Eyes with a MH duration >6 months or with a MH >1,000 *μ*m in diameter were excluded. Patients with other ocular diseases, such as those with an epiretinal membrane (ERM), macular edema, rhegmatogenous retinal detachment, glaucoma, diabetic retinopathy, uveitis, and high pathological myopia were also excluded.

All of the procedures adhered to the tenets of the Declaration of Helsinki. The protocol of this study was approved by the Institutional Review Board and Ethics Committee of Matsumoto Dental University, and a written informed consent for the examination and surgery was obtained from all of the patients.

### 2.2. Surgical Procedures

The vitreoretinal surgery was performed with standard 23-gauge (23G; *n* = 3) or 25G (*n* = 35) instruments by a single surgeon (KO). After core vitrectomy, a posterior vitreous detachment (PVD) was created by suction with the vitrectomy cutter after TA (Kenakolt-A; Bristol Pharmaceuticals KK, Tokyo, Japan or MaQaid, Wakamoto Pharmaceuticals Co., Ltd., Tokyo, Japan) was injected into the vitreous cavity [19, 20]. A PVD already existed in 6 eyes with stage 4 MH. Because nuclear cataracts can develop after PPV, 36 eyes underwent PPV combined with phacoemulsification and aspiration (PEA) with implantation of an intraocular lens [21]. Two of the 38 eyes were already pseudophakic. In the end, all 38 eyes were pseudophakic after the PPV.

During the MH surgery, the ILM of all eyes was made more visible with TA. The ILM was additionally stained with brilliant blue G (Brilliant Peel®, Geuder, Heidelberg, Germany) in 9 eyes. The ILM was grasped at the temporal raphe to avoid damaging the retinal nerve fiber layer in the thicker nasal, superior, and inferior areas. The size of the peeled area was measured with the ImageJ software in the video images recorded during the surgery. Then fluid-air exchange was performed with the air pressure set at 35 mmHg. At the completion of the surgery, 20% sulfur hexafluoride (SF_6_) was injected for a gas tamponade. The patients were instructed to remain in a prone position for at least 7 days.

### 2.3. Spectral-Domain Optical Coherence Tomography (SD-OCT)

SD-OCT examinations were performed with the Spectralis HRA + OCT (Heidelberg Engineering, Heidelberg, Germany) before and at 1, 3, 6, 12, and 24 months after the PPV. The horizontal and vertical images centered on the fovea in the cross-hair mode (30°) were evaluated. Circular scans of 1.8 mm radius centered on the optic disc were recorded to measure the RNFL thickness. Each OCT image was obtained along a 360-degree path around the optic disc. The mean RNFL thickness of the 1,536 measured points was called the mean global thickness. The mean RNFL thickness of the temporal-superior (1° to 45°), temporal (46° to 135°), temporal-inferior (136° to 180°), nasal-inferior (181° to 225°), nasal (226° to 315°), and nasal-superior (316° to 360°) sectors was obtained for the sector analyses. During the follow-up visits, the baseline circumpapillary scan was set as the reference scan, and the same peripapillary region was scanned with the eye tracking system functioning.

### 2.4. Statistical Analyses

The results are expressed as the means ± standard deviations (SDs). The best-corrected visual acuity (BCVA) was measured with a Landolt C chart, and the decimal values were converted to logarithm of the minimal angle of resolution (log⁡MAR) units. Data were analyzed with the SPSS for Windows software (version 11.0J, SPSS, Chicago, IL). The significance of differences in the pre- and postoperative values was determined by paired *t*-tests. The significance of differences between the eyes with a closed MH and the fellow eyes in 36 unilateral cases was determined by unpaired *t*-tests. A difference was considered to be statistically significant when the *P* was <0.05.

## 3. Results

### 3.1. Demographics and Best-Corrected Visual Acuity of All Eyes

The clinical characteristics of all of the eyes are summarized in Table 1. The MH was closed in all of the eyes after the first operation. The postoperative BCVA was −0.003 ± 0.169log⁡MAR units which was significantly better than the preoperative BCVA at 0.472 ± 0.256log⁡MAR units (*P* < 0.00001). During the follow-up period, an aftercataract developed in three eyes of 3 patients, and neodymium:yttrium aluminum-garnet (Nd:YAG) laser capsulotomy was performed.

### 3.2. Comparisons of Preoperative Global and Sectoral RNFL Thicknesses of Operated and Fellow Eyes

The preoperative peripapillary global and sectoral RNFL thicknesses of the experimental and fellow eyes are shown in Table 2. Two eyes were excluded because a MH was present bilaterally. The mean RNFL thickness in the MH eyes was thicker than that of the fellow eyes in the temporal-superior and nasal-inferior sectors (127.0 ± 15.3 *μ*m versus 121.6 ± 22.8 *μ*m and 109.6 ± 25.7 *μ*m versus 103.5 ± 21.7 *μ*m, resp.). However, the differences were not significant. The preoperative global and sectoral RNFL thicknesses were not significantly different between the operated and fellow eyes.

### 3.3. Longitudinal Changes in Global and Sectoral RNFL Thicknesses

The global and sectoral RNFL thicknesses of the preoperative and 1, 3, 6, 12, and 24 months after MH surgery are shown in Table 3. Unfortunately, some patients did not visit at all times except at 12 months. Thus, the number of examined eyes was only twenty-two. The mean global RNFL was significantly thicker at 1 month (101.1 ± 11.2 *μ*m, *P* < 0.0001), 3 months (98.4 ± 11.3 *μ*m, *P* < 0.01), and 6 months (97.1 ± 10.4 *μ*m, *P* < 0.05) than the preoperative value (93.9 ± 9.1 *μ*m). At 12 and 24 months, the global RNFL thickness was not significantly different from the preoperative thickness (95.2 ± 10.9 *μ*m and 93.4 ± 11.4 *μ*m, resp.).

The mean RNFL of the temporal-superior and temporal-inferior sectors were significantly thicker at 1 month (*P* < 0.0001, *P* < 0.0001, resp.), 3 months (*P* < 0.0001, *P* < 0.001, resp.), and 6 months (*P* < 0.0001, *P* < 0.01, resp.) than the preoperative thickness. In addition, the mean RNFL thickness of the temporal-superior sector was still significantly thicker at 12 months (*P* < 0.05).

The mean RNFL of the nasal-superior sector was significantly thicker at 1 month (*P* < 0.0001), 3 months (*P* < 0.01), 6 months (*P* < 0.05), and 12 months (*P* < 0.05). These findings were similar to that for the temporal-superior sector.

The mean RNFL of the temporal and nasal sectors was significantly thicker at 1 month (*P* < 0.001) and 3 months (*P* < 0.001, *P* < 0.05, resp.) after the MH surgery. The mean RNFL thickness did not differ significantly at 6, 12, and 24 months from baseline thickness.

In contrast to these findings, the mean RNFL thickness of the nasal-inferior sector did not change significantly at 1, 3, and 6 months from that of the preoperative thickness. On the other hand, the mean RNFL was thinner at 12 and 24 months although the difference was not significant. The thicknesses of the global and all of the sectors were not significantly decreased at 24 months.

The mean intraocular pressure (IOP) at each time point is presented in Table 3. The IOP was significantly lower at 6 and 12 months after the MH surgery. On the other hand, the IOP was not significantly changed at 1 and 3 months when the global and sectoral RNFL were significantly thicker.

### 3.4. RNFL Thicknesses at 12 Months after MH Surgery

The RNFL thickness of 36 eyes was measured at 12 months after surgery (Table 4). The mean RNFL was significantly thicker at the temporal-superior, temporal-inferior, and nasal-superior sectors than the preoperative thicknesses of the corresponding sectors (130.7 ± 17.2 *μ*m versus 126.9 ± 17.6 *μ*m, *P* < 0.01; 141.1 ± 19.8 *μ*m versus 136.3 ± 18.1 *μ*m, *P* < 0.01; and 104.1 ± 22.3 *μ*m versus 101.1 ± 19.5 *μ*m, *P* < 0.05; paired *t*-tests). Only the mean RNFL in the nasal-inferior sector was thinner than the preoperative thickness although the difference was not significant.

## 4. Discussion

Our results showed that the RNFL was significantly thicker in all sectors but the nasal-inferior sector at 1 and 3 months after PPV with TA-assisted ILM peeling. The increase was still present at 6 months postoperatively except in the temporal and nasal sectors. At 12 months, the increase in the thickness in the temporal-superior and nasal-superior or temporal-inferior sectors was still present.

Kurimoto et al. reported that the temporal quadrant was significantly thinner in MH eyes especially in eyes with stage 3 MH [22], but it has been reported that there were no changes in the RNFL thickness in long standing MHs such as those at stage 4 [23]. Our results showed that there were no significant differences in the RNFL thicknesses between the eyes with MH and fellow eyes preoperatively. In the earlier reports, the RNFL thickness was measured by scanning laser polarimeter and not by SD-OCT. The RNFL thickness was measured within a 10-pixel-wide ellipse that was concentric with the optic disc and was 1.5 disc diameter in size [22]. On the other hand, circular scans of 1.8 mm radius were performed by SD-OCT in this study. This difference of scan area may explain the discrepancies between the previous report and our results.

It has been reported that the RNFL was thicker in the temporal sector at the baseline in eyes with an ERM and was thinner after successful surgery [24]. However, the RNFL thickness was evaluated in not only MH eyes but also some eyes with a MH and ERM in some studies [16, 18, 25–27]. We believe it is important to show that there was no significant difference in the preoperative RNFL thickness between the eyes with a MH and the fellow eyes.

A transient increase of the RNFL thickness after MH surgery was recently reported [14–16]. The global or mean overall thickness was increased at 1 month, and it returned to preoperative values at 3 or 6 months [14–16]. An increase at 1 month is in agreement with our results. However, a residual increase at not only 3 months but also 6 and 12 months in some sectors was the only change in our study. In contrast, it has been reported that the global RNFL thickness was significantly decreased at 6 months [16] which is contrary to the findings in our patients. There are several possible reasons for this discrepancy. First, 23 of the 30 patients in the earlier study had an ERM and 6 had a MH [16]. In eyes with an ERM, the RNFL in the temporal quadrant was significantly thicker at the baseline although the global thickness was not significantly thicker than that of the fellow eyes [24]. The RNFL in the temporal pathological area of eyes with an ERM was significantly thinner at 3, 6, and 12 months postoperatively. The longitudinal changes in the RNFL thickness compared to the preoperative values after ERM surgery must differ from that in eyes with a MH. In contrast, the RNFL thickness of only MH cases was measured by Toba and Hibi. They found no significant decrease in temporal sector after MH surgery. Instead, an increase in the RNFL thickness in the nasal quadrant or a decrease in the nasal-inferior sector was observed [14, 15].

The second possible reason for the discrepancy is the differences in the SD-OCT instruments used. In recent studies, the SD-OCT instruments used for RNFL thickness measurements were the Cirrus OCT (Carl Zeiss Medlitec, Dublin, CA) [14, 17, 18] or Spectralis HRA + OCT [15, 16]. The reproducibility of the RNFL thickness measurements with the Spectralis SD-OCT with the eye tracking system is excellent for the follow-up measurements [28, 29]. Thus, smaller changes in the RNFL thickness can be detected by Spectralis SD-OCT compared to the Cirrus OCT. We believe that the eye tracking system was not present in the Cirrus OCT at that time [14, 17, 18]. This may account for the discrepancies.

Third, phacovitrectomy may contribute to the significant increases in the RNFL thicknesses at 6 and 12 months. When cataract surgery is not performed during the PPV, cataract surgery was needed in 6 of 13 MH eyes within a year [18] or all 8 eyes by 2 years [27]. The development of cataract may have some influence on the measurements of RNFL thickness. In contrast, all 38 eyes were pseudophakic during follow-up period in our study. Thus, our measurements of the RNFL thickness were probably more reliable.

Toba et al. measured the RNFL thickness by the same type of SD-OCT instrument, the Spectralis HRA + OCT, as we did [15]. The surgical procedures were also similar to our procedures. They compared the effects of ICG-, BBG-, and TA-assisted ILM peeling on the RNFL thickness. In spite of the use of these agents, the changes in the RNFL thickness did not differ from what we found for up to 12 months, for example, the postoperative increase of the global and sectorial thicknesses except the nasal-inferior thickness at only 1 month after surgery. Because the mean RNFL thicknesses were not given in their text, it was difficult to compare the absolute values to ours. However, the pattern of longitudinal changes was similar to our results. There were no significant differences at 3, 6, and 12 months after surgery in our study, and the changes in their RNFL thicknesses appeared similar to ours.

The RNFL sectors that increased or decreased postoperatively differed in the different studies. At 6 months, the nasal RNFL was significantly thicker in only the study by Hibi et al. [14]. On the other hand, the inferior RNFL was reduced at 12 months as reported by Lalezary et al. [18] or at 24 months by Thinda et al. [27]. The inferior sector is the 226–315° quadrant with the Cirrus OCT. When Spectralis OCT was used, this sector can be divided into the nasal-inferior and temporal sectors. A significant decrease at 6 and 12 months after surgery in the temporal-inferior sector was reported by Toba et al. [15]. We similarly found that the RNFL thickness in only the temporal-inferior sector did not increase even at 1 and 3 months. In addition, it decreased at 12 and 24 months but the decrease was not significant.

The exact mechanism causing the transient increase in the RNFL thickness after MH surgery has been investigated. There are many studies that suggest that indocyanine green (ICG), which was used to make the ILM more visible, has a toxic effect on the inner retina [10, 30, 31]. However, there was no significant decrease of the RNFL thickness in the ICG group when the ICG concentration was low and was washed out quickly [15]. In our cases, we can exclude the toxic effect of ICG because we did not use it.

Another possible mechanism for the transient increase in the RNFL thickness was the retinal damage induced during the infusion of saline and/or the air that were directed against the retinal surface from the cannula during vitrectomy [14]. They used 20G instruments in 17 of 20 eyes while we used only 23G or 25G instruments. In the 20G system, the infusion cannula is usually placed at the temporal-inferior region and is aimed toward the optic disc or nasal side of the retina. With the 23G or 25G instruments, the infusion cannula is generally placed obliquely, and the area of the retina where the saline or air is directed must be different. This difference may be associated with the sector that is increased or decreased.

We cannot explain why the RNFL thickness in only the nasal-inferior sector decreased [15] or did not increase in our patients. More recently, a thinning of the inferior RNFL was observed at 12 months, and they suggested that this was due to early glaucomatous damage [18]. However, we did not find such a decrease or elevation of the IOP in this study. Instead, we believe that the surgical procedures, such as creating the posterior vitreous detachment, grasping the ILM with forceps, or making an incision by microknife, and attachment to the retina during ILM peeling must differ. So these surgical factors may be related to the changes of the RNFL thickness by the loss of retinal ganglion cell.

There are several limitations to this study. First, this was a retrospective study and the number of patients studied was small which may have affected the reliability of the statistical analyses. Second, a single surgeon performed all of the surgeries which may limit any broad conclusions. Third, functional analysis such as visual field analysis, microperimetry, and electroretinography were not performed. Further prospective studies will be necessary to confirm our results.

In conclusion, the RNFL thickness in all but the nasal-inferior sector increased significantly at 1, 3, 6, and 12 months after MH surgery combined with cataract surgery. The transient increase in the RNFL thickness may be due to a mild edema of the inner retinal layer cause by the procedures used in the MH surgery. However, the exact mechanism was not definitively determined. Further studies including a functional analysis are needed.

## Figures and Tables

**Table 1:** Demographics of study population.

Number of eyes	38
Number of patients	37
Age, (yrs)	67.2 ± 6.4 (51–76)
Sex (men/women)	17/20
Axial length, mm	23.78 ± 0.89 (22.64–25.61)
Macular hole stage	Stage 2; 16, stage 3; 16, stage 4; 6
Mean macular hole size, *µ*m	376.3 ± 152.1 (160–674)
Mean visual acuity, log⁡MAR unit	
Preoperative	0.472 ± 0.256
Postoperative	−0.003 ± 0.169
ILM staining	
TA only	29
TA + BBG	9
MIVS	23G; 3, 25G; 35
Combined with PEA/IOL/pseudophakia	36/2

BBG = brilliant blue G; MIVS = microincision vitreous surgery; PEA/IOL = phacoemulsification and intraocular lens implantation; TA = triamcinolone acetonide.

Data are the mean ± standard deviation.

**Table 2:** Comparison of circumpapillary global and sectoral RNFL thickness (*µ*m) in affected and fellow eyes (*n* = 36).

Sector	Affected eye	Fellow eye	*P* ^*∗*^
Global	95.0 ± 10.4	92.6 ± 11.5	0.36
Temporal-superior	127.0 ± 15.3	121.6 ± 22.8	0.24
Temporal	70.5 ± 10.3	69.1 ± 9.4	0.57
Temporal-inferior	137.6 ± 17.4	140.8 ± 13.6	0.39
Nasal-superior	102.1 ± 19.5	103.8 ± 22.4	0.73
Nasal	70.0 ± 17.9	66.3 ± 19.1	0.40
Nasal-inferior	109.6 ± 25.7	103.5 ± 21.7	0.28

Data are the mean ± standard deviation.

^*∗*^Unpaired *t*-test.

**Table 3:** Comparison of circumpapillary global and sectoral RNFL thickness (*µ*m) and intraocular pressure (mmHg) during 24-month follow-up period (*n* = 22).

Sector	Preoperative	Postoperative				
		1 month	3 months	6 months	12 months	24 months
Global	93.9 ± 9.1	101.1 ± 11.2^§^	98.4 ± 11.3^†^	97.1 ± 10.4^*∗*^	95.2 ± 10.9	93.4 ± 11.4
TS	125.1 ± 17.1	135.2 ± 17.5^§^	132.1 ± 16.9^§^	131.2 ± 15.1^§^	129.7 ± 17.5^*∗*^	126.7 ± 17.5
T	70.7 ± 11.3	78.3 ± 10.5^‡^	76.2 ± 9.0^‡^	73.6 ± 10.6	71.3 ± 18.6	72.1 ± 11.0
TI	137.7 ± 16.0	148.6 ± 19.1^§^	144.0 ± 17.0^‡^	143.1 ± 16.3^†^	141.1 ± 18.3	136.6 ± 18.7
NS	99.1 ± 20.7	107.1 ± 23.8^§^	105.0 ± 24.1^†^	104.3 ± 24.4^*∗*^	103.3 ± 24.8^*∗*^	99.8 ± 25.8
N	68.5 ± 14.6	74.1 ± 18.9^‡^	72.2 ± 17.6^*∗*^	71.1 ± 16.8	69.4 ± 17.1	67.4 ± 17.6
NI	107.4 ± 20.7	112.0 ± 19.9	107.7 ± 20.0	107.2 ± 18.6	105.0 ± 18.5	103.5 ± 19.9
Mean IOP	14.7 ± 2.4	14.0 ± 2.9	13.6 ± 2.4	13.7 ± 2.3^*∗*^	13.6 ± 1.8^†^	14.7 ± 2.5

TS = temporal-superior; T = temporal; TI = temporal-inferior; NI = nasal-inferior; N = nasal; NS = nasal-superior.

IOP = intraocular pressure.

Data are the mean ± standard deviation.

^*∗*^ *P* < 0.05.

^†^ *P* < 0.01.

^‡^ *P* < 0.001.

^§^ *P* < 0.0001.

Paired *t*-test compared with preoperative value.

**Table 4:** Comparison of circumpapillary global and sectoral RNFL thickness (*μ*m) at 12 months after surgery (*n* = 38).

Sector	Before operation	12 months postoperatively	*P* ^*∗*^
Global	96.4 ± 13.3	96.3 ± 11.3	0.97
Temporal/superior	126.9 ± 17.6	130.7 ± 17.2	0.0049
Temporal	70.7 ± 12.2	72.6 ± 12.2	0.12
Temporal/inferior	136.3 ± 18.1	141.1 ± 19.8	0.0035
Nasal/superior	101.1 ± 19.5	104.1 ± 22.3	0.044
Nasal	68.6 ± 17.8	69.6 ± 17.9	0.41
Nasal/inferior	107.6 ± 25.4	105.1 ± 21.5	0.36

Data are the mean ± standard deviation.

^*∗*^Paired *t*-test.
